# Evaluation of an Automatic Classification Algorithm Using Convolutional Neural Networks in Oncological Positron Emission Tomography

**DOI:** 10.3389/fmed.2021.628179

**Published:** 2021-02-26

**Authors:** Pierre Pinochet, Florian Eude, Stéphanie Becker, Vijay Shah, Ludovic Sibille, Mathieu Nessim Toledano, Romain Modzelewski, Pierre Vera, Pierre Decazes

**Affiliations:** ^1^Department of Nuclear Medicine, Henri Becquerel Cancer Center, Rouen, France; ^2^LITIS Quantif-EA 4108, University of Rouen, Rouen, France; ^3^Siemens Medical Solutions USA, Inc., Knoxville, TN, United States

**Keywords:** positron emission tomography, convolutional neural network, diffuse large B cell lymphoma (DLBCL), artificial intelligence-AI, fluorodeoxyglucose (^18^F-FDG)

## Abstract

**Introduction:** Our aim was to evaluate the performance in clinical research and in clinical routine of a research prototype, called positron emission tomography (PET) Assisted Reporting System (PARS) (Siemens Healthineers) and based on a convolutional neural network (CNN), which is designed to detect suspected cancer sites in fluorine-18 fluorodeoxyglucose (^18^F-FDG) PET/computed tomography (CT).

**Method:** We retrospectively studied two cohorts of patients. The first cohort consisted of research-based patients who underwent PET scans as part of the initial workup for diffuse large B-cell lymphoma (DLBCL). The second cohort consisted of patients who underwent PET scans as part of the evaluation of miscellaneous cancers in clinical routine. In both cohorts, we assessed the correlation between manually and automatically segmented total metabolic tumor volumes (TMTVs), and the overlap between both segmentations (Dice score). For the research cohort, we also compared the prognostic value for progression-free survival (PFS) and overall survival (OS) of manually and automatically obtained TMTVs.

**Results:** For the first cohort (research cohort), data from 119 patients were retrospectively analyzed. The median Dice score between automatic and manual segmentations was 0.65. The intraclass correlation coefficient between automatically and manually obtained TMTVs was 0.68. Both TMTV results were predictive of PFS (hazard ratio: 2.1 and 3.3 for automatically based and manually based TMTVs, respectively) and OS (hazard ratio: 2.4 and 3.1 for automatically based and manually based TMTVs, respectively). For the second cohort (routine cohort), data from 430 patients were retrospectively analyzed. The median Dice score between automatic and manual segmentations was 0.48. The intraclass correlation coefficient between automatically and manually obtained TMTVs was 0.61.

**Conclusion:** The TMTVs determined for the research cohort remain predictive of total and PFS for DLBCL. However, the segmentations and TMTVs determined automatically by the algorithm need to be verified and, sometimes, corrected to be similar to the manual segmentation.

## Introduction

Positron emission tomography (PET) with fluorine-18 (^18^F) fluorodeoxyglucose (FDG) has an important contribution in the diagnosis and the management of oncological pathologies by highlighting regions with a high glucidic metabolism (1).

PET can establish an initial staging of tumor lesions (2), enable treatment optimization, and evaluate treatment effectiveness or possible relapse (3–8). It also provides prognostic parameters in certain types of cancer, in particular in onco-hematology, such as the Deauville score, which evaluates the therapeutic response and is used in clinical routine, or the total metabolic tumor volume (TMTV) (9).

TMTV represents, generally on FDG PET, the volume of the entire cancerous disease. It is obtained by segmenting each diagnosed lesion. TMTV has been shown to be an independent prognostic factor in lymphoma (10). Recently, Albano et al. have shown its predictive nature on progression-free survival (PFS) in elderly Hodgkin's lymphoma (11) and mantle cell lymphoma (12), but also on total and PFS in Burkitt lymphoma (13) and cerebral lymphoma (14). However, this parameter has some limitations. The first is that the measurement is time-consuming to make, explained by the fact that each lesion must be segmented individually, a task that cannot be performed manually in clinical practice. The second is the absence of a standard method for the segmentation of hypermetabolic lesions, which is responsible for some variability in the determination of TMTV. Thus, a fixed threshold of SUV_max_ (for example 41% for lymphomas) for each lesion is frequently used (15). However, this may not be appropriate for all pathological foci, particularly in the case of heterogeneous tumor fixation and adjacent physiological volume with high uptake (16).

A problem frequently encountered during the interpretation and segmentation of the images is differentiating between benign physiological (e.g., brain, heart, liver, kidney, and bladder) or inflammatory foci, and pathological foci suspicious for cancerous lesions. This is particularly true for malignant tumors with a low avidity for glucose, unusual location, or small size or in the presence of attenuation and/or motion artifacts (17). Moreover, inflammatory or infectious foci, or even foci with a high physiological consumption of glucose may have a sufficiently high FDG uptake to make it not possible to eliminate a cancerous origin (18, 19).

Intra- and interobserver interpretation of FDG PET/computed tomography (CT) findings has a high level of agreement in studies involving single site and experienced readers for lymphoma, lung, and head and neck cancers (20–22). Widespread adoption of TMTV would be facilitated by tools to assist image interpretation and standardize results. Automatic segmentation has also proven to be a prerequisite for certain studies, particularly in the field of radiomics.

In recent years, several automatic segmentation methods have been developed. They can be divided into two main groups. The first is based on an ROI placed manually by the physician within which a threshold relative to SUV_max_ is applied (23–25). The resulting segmentation depends on the defined ROI and is generally not optimal. A second approach, which is less time-consuming and observer-independent, uses supervised machine learning to analyze PET/CT images (26). A research software prototype called PET Assisted Reporting System (PARS), based on convolutional neural networks (CNNs), has recently been developed by Siemens Healthineers to classify hypermetabolic foci into benign and malignant and to provide parameters such as TMTV, total lesion glycolysis (TLG), and Deauville score (27). With this algorithm, PET volumes of interest are first segmented by using a fixed thresholding algorithm. Each volume of interest is then evaluated independently by using a combination of PET and CT multiplanar reconstructions, PET maximum intensity projections (MIPs), and atlas positions to predict the anatomic localization of FDG foci. These are input to a CNN that determines whether a focus is suspicious for malignancy. The training and validation sets were carried out on cohorts of patients with either lung cancer or lymphoma. A first, internal evaluation of this tool showed good accuracy of the automatic segmentation of FDG positive foci, and also good sensitivity and specificity of the classification in staging patients with lung cancer and lymphoma compared with manual segmentation (27).

The aim of this study was to verify the performance of PARS in order to determine its usefulness in research and clinical routine.

## Method

### Study Design

This retrospective monocentric study included patients treated at the Henri Becquerel Cancer Center, Rouen, France. Two patient cohorts were analyzed: a first clinical research cohort composed of patients with diffuse large B-cell lymphoma (DLBCL), as TMTV is a well-known prognostic factor for this disease (10), and a second clinical routine cohort composed of patients selected at random and followed up for miscellaneous cancers to evaluate if an automatic measurement of TMTV can be performed in routine. All patients were over 18 years of age. The baseline PET/CT was analyzed for the DLBCL clinical research cohort. For the routine clinical cohort, including patients with suspected or confirmed cancer, a baseline or a follow-up PET/CT was analyzed. The study was approved by the institutional review board (no. 1901B). Patients were informed about the use of anonymized data for research and their right to oppose this use. Fully anonymized data were used, and explicit consent was waived.

### Research Cohort

Concerning the research cohort, 119 patients followed up for DLBCL were included between November 2004 and September 2014, and their initial FDG PET/CT was analyzed.

PET/CT scans were acquired on a Biograph 16 (Siemens Healthineers, Knoxville, TN, USA). Patients fasted for at least 4 h and were injected with FDG at an activity of 3.5 MBq/kg of body weight. Images were acquired 60 min after injection at 2.5 min per bed position. The manual segmentation of lesions was performed using semiautomatic software (Planet Onco, version 2.0, DOSIsoft®, Cachan, France). A volume of interest was set around each lesion on the PET images. Then a fixed threshold value of 41% of SUV_max_ was applied to define the volume for each segmented lesion. The volumes of all suspicious lesions in a particular patient were added to compute the TMTV. The manual segmentation was performed by two nuclear physicians for each patient (MT and FE). One of the manual segmentations (MT), chosen arbitrarily, was used for the calculation of the Dice scores. The average of the two TMTVs was used for all other calculations.

Five-year follow-up, including PFS and overall survival (OS), was available for this cohort.

### Routine Cohort

Concerning the routine cohort, 430 patients referred for cancer assessment underwent routine thoraco-abdomino-pelvic or whole body PET/CT (according to the indication), and with at least one tumoral uptake, were included between August 2018 and February 2020.

PET/CT scans were acquired on GE 710 (General Electric, Milwaukee, WI, USA) or Biograph Vision 600 (Siemens Healthineers, Knoxville, TN, USA). Patients fasted for at least 4 h and were injected with FDG at a dose of 3.0 MBq/kg of body weight. Images were acquired 60 min after injection at 2 min per bed position (GE 710) or by continuous bed motion (Biograph Vision).

The manual segmentation of lesions was performed using another semiautomatic software (PET VCAR, General Electric®) during routine clinical activity by two different nuclear medicine physicians (PD and PP). A volume of interest was set around each lesion on the PET images according to an adaptive thresholding (28), manually adapted if necessary according to medical advice. After the database was gathered, a second reading was done in order to check and confirm the suspicious character of the different segmented foci. These values were added to compute the TMTV.

Data of the two cohorts of patients are summarized in Table 1.

**Table 1:** Summary results from two patient cohorts.

**Cancer type**	**Number**	**Frequency** ** (%)**	**Mean Dice** ** score**	**Median Dice** ** score**	**Minimal Dice** ** score**	**Maximal Dice** ** score**	**ICC** ** (*p*-value)**
Clinical research database							
Lymphoma (DLBCL)	119	100	0.52	0.65	0	0.93	0.68 (*p* < 0.001)
Clinical routine database							
Lung	150	34.88	0.51	0.59	0	0.94	0.71 (*p* < 0.001)
Lymphoma	71	16.51	0.40	0.45	0	0.97	0.56 (*p* < 0.001)
Breast	28	6.51	0.21	0	0	0.87	
Unknown	26	6.05	0.30	0.04	0	0.93	
Colorectal	25	5.81	0.46	0.56	0	0.88	
Melanoma	23	5.35	0.37	0.50	0	0.85	
Head and neck	23	5.35	0.40	0.36	0	0.93	
Esophagus	19	4.42	0.54	0.74	0	0.90	
Ovary	8	1.86	0.51	0.61	0	0.94	
Anal	5	1.16	0.10	0	0	0.46	
Kidney	5	1.16	0.24	0.22	0	0.61	
Sarcoma	5	1.16	0.22	0	0	0.63	
Carcinoma of unknown primary (CUP)	4	0.93	0.28	0.30	0	0.51	
Cervix	4	0.93	0.16	0	0	0.64	
Endometrium	4	0.93	0.18	0.02	0	0.66	
Pancreas	4	0.93	0.35	0.27	0	0.86	
Prostate	4	0.93	0.24	0.07	0	0.82	
Pleural	3	0.70	0.21	0	0	0.63	
Adrenal	3	0.70	0.18	0	0	0.54	
Testis	3	0.70	0.19	0	0	0.56	
Skin	2	0.47	0.40	0.40	0	0.79	
Stomach	2	0.47	0.42	0.42	0.03	0.81	
Myeloma	2	0.47	0.43	0.43	0	0.87	
Bladder	2	0.47	0.85	0.85	0.85	0.85	
Liver	1	0.23	0.53	0.53	0.53	0.53	
Leukemia	1	0.23	0.74	0.74	0.74	0.74	
Paraganglioma	1	0.23	0.70	0.70	0.70	0.70	
Thymus	1	0.23	0.94	0.94	0.94	0.94	
Thyroid	1	0.23	0.20	0.20	0.20	0.20	
Miscellaneous	430	100.00	0.42	0.48	0	0.97	0.61 (*p* < 0.001)

*DLBCL, diffuse large B-cell lymphoma*.

### Convolutional Neural Network Use

PET/CT images were analyzed using a software prototype called PARS (Siemens Healthineers, Knoxville, TN, USA). A cylindrical reference region was automatically placed in the center of descending thoracic aorta to measure the mean blood pool uptake (SUV_BP_). Regions on PET images with SUV_peak_ greater than SUV_BP_ + 2 std_SUVBP_ were identified and segmented using 42% of local SUV_max_. Only segmentations with volumes over 2 ml (research cohort) or 1 ml (routine cohort) were selected to be processed by the CNN, which specifies location and physiological or suspicious character of the different foci.

### Statistical Analysis

For both cohorts, agreement between automatic and manual segmentations was characterized using the Dice score. Differences between TMTVs from PARS and manual segmentation were determined using intraclass correlation coefficient (ICC), notably for subgroups of more than 30 patients. Comparisons were also made by way of Bland–Altman plots.

The prognostic value for PFS and OS for both automatic and manual TMTVs was analyzed in the research cohort. Hazard ratios were calculated on continuous data. Receiver operating characteristic (ROC) curves were used to determine TMTV cutoff thresholds by Youden's index. Survival functions were computed by Kaplan–Meier analyses and used to estimate survival time statistics for low and high TMTV groups with log-rank tests.

## Results

### Research Cohort

**Figure 1:**
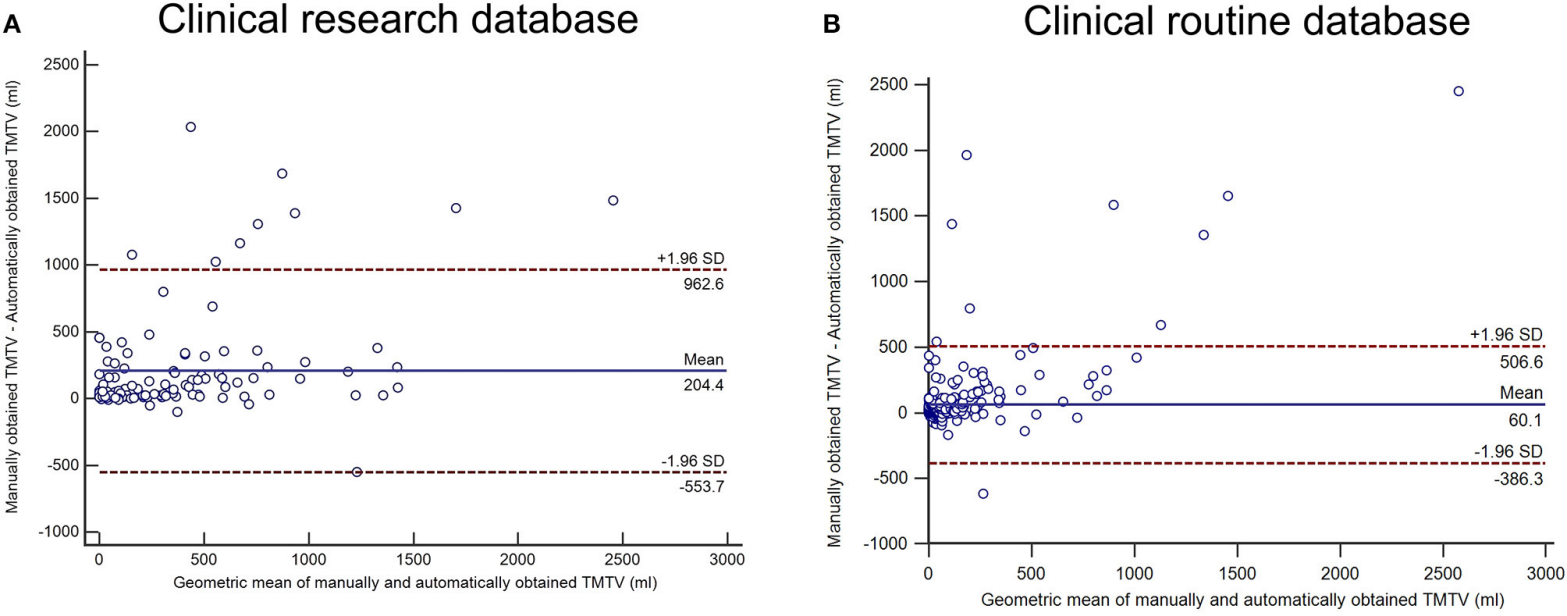
Bland–Altman analysis between manually and automatically obtained total metabolic tumor volumes (TMTVs) for the clinical research cohort **(A)** and the clinical routine database **(B)**.

Concerning the research cohort, 119 patients were included in the analysis. The median age was 65.8 years. Ninety-three patients had stage 3 or 4 DLBCL according to the Ann Arbor classification. Thirty received first-line treatment with R-ACVBP (doxorubicin, cyclophosphamide, vindesine, bleomycin, prednisone regimen) and 89 with R-CHOP (cyclophosphamide, doxorubicin, vincristine, and prednisone regimen). The ICC between the two manual TMTVs was 0.86 (*p* < 0.001), confirming the reproducibility of the segmentations. The median Dice score across all patients between the set of PARS ROI's labeled as suspicious and the set of manual ROI's was 0.65. The average Dice score was 0.52. The median TMTV_PARS_ was 194.79 ml, maximum 1,821 ml, and minimum 0 ml. The median TMTV_manual_ was 313.34 ml, maximum 3,304 ml, and minimum 8 ml (Table 1 and Supplementary Figure 1). The ICC between PARS and manual TMTVs was 0.68 (Table 1). Concerning the Bland–Altman plot, the deviation from the mean between TMTV_manual_ and TMTV_PARS_ was +204 ml with a confidence interval of −554 to +963 ml (see Figure 1A).

After a median follow-up of 5 years, 60 patients presented a recurrence of the disease and 54 deceased. The 5-year survival rates were 49.6% for PFS and 54.6% for OS.

**Figure 2:**
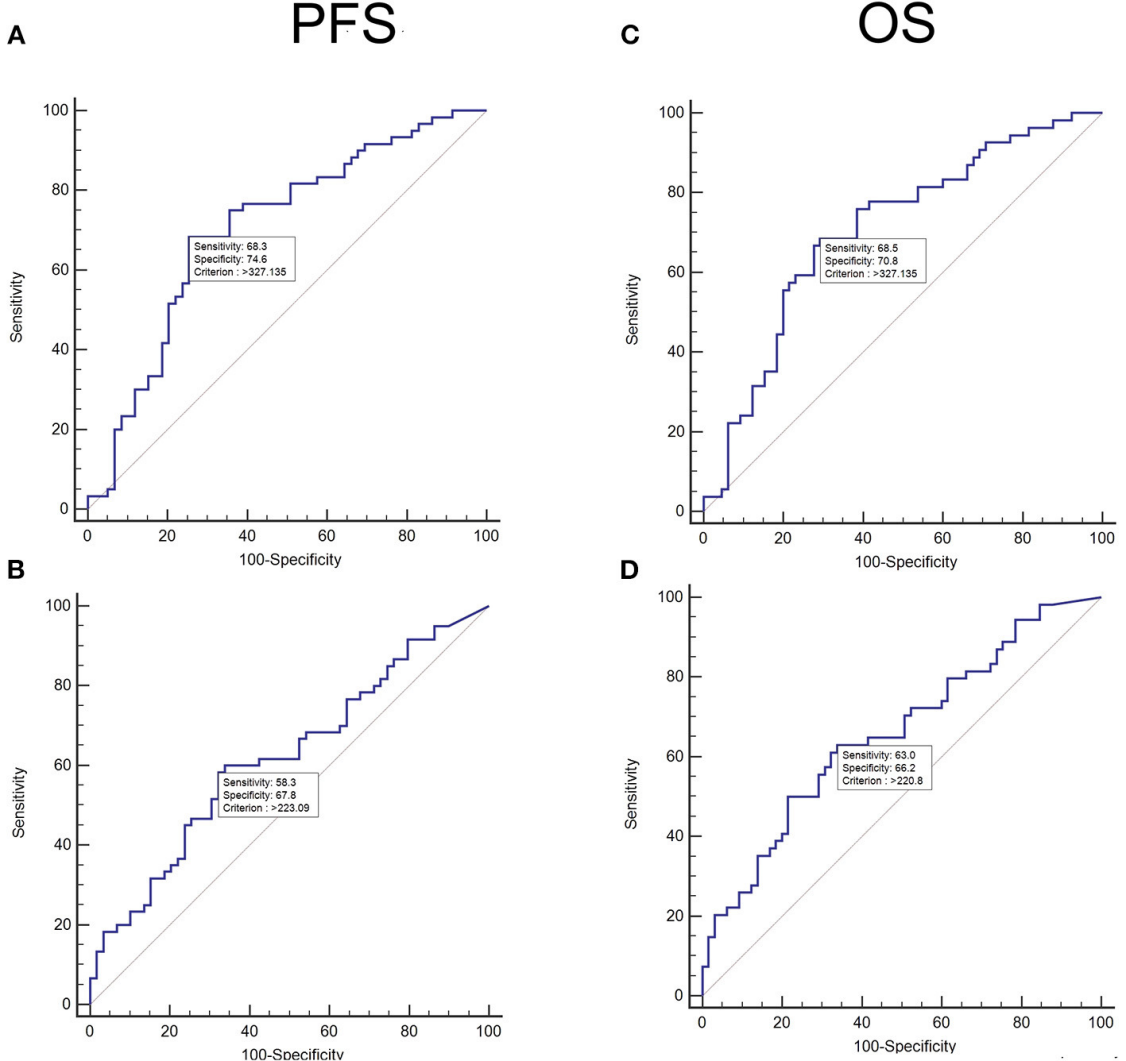
Receiver operating characteristic (ROC) curve analysis of the population of diffuse large B-cell lymphomas (clinical research database) for progression-free survival (PFS) for manually obtained total metabolic tumor volumes (TMTVs) **(A)** and automatically obtained TMTVs **(B)** and for overall survival (OS) for manually obtained TMTVs **(C)** and automatically obtained TMTVs **(D)**.

**Figure 3:**
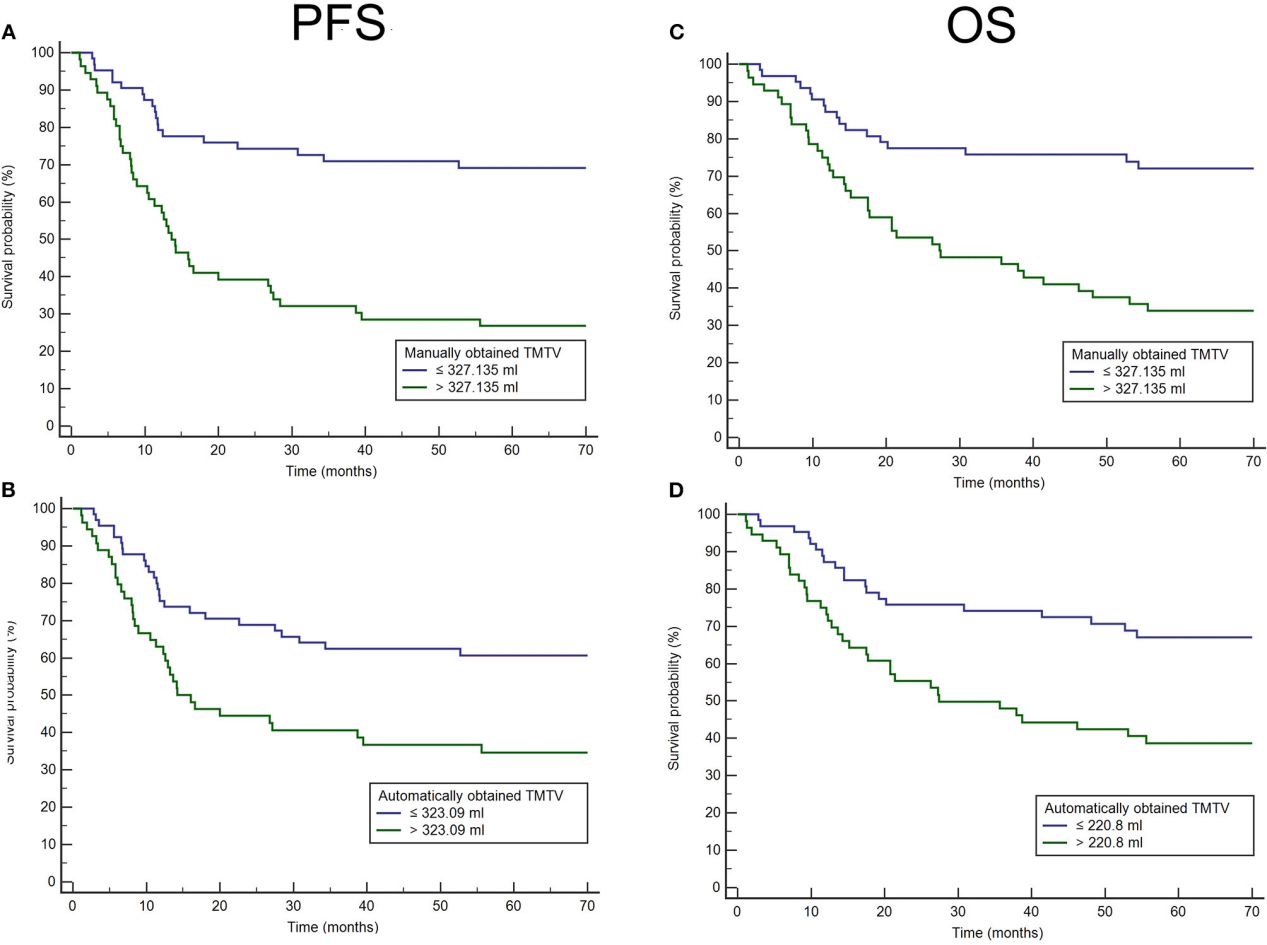
Kaplan–Meier analysis of the population of diffuse large B-cell lymphomas (clinical research database) for progression-free survival (PFS) for manually obtained total metabolic tumor volumes (TMTVs) **(A)** and automatically obtained TMTVs **(B)** and for overall survival (OS) for manually obtained TMTVs **(C)** and automatically obtained TMTVs **(D)**.

The area under the ROC curve for predicting PFS was 0.62 for TMTV_PARS_ and 0.71 for TMTV_manual_ (Figures 2A,B). The optimal cutoffs for predicting PFS were 223.09 ml for TMTV_PARS_ and 327.14 ml for TMTV_manual_. The 5-year PFS rates were 61.5 and 35.2% for the low- and high-TMTV_PARS_ groups and 69.8% and 26.8% for the low- and high-TMTV_manual_ groups, respectively (Figures 3A,B). The log-rank test indicated a significantly longer PFS time in the low-TMTV group for both TMTV estimation methods (*p* = 0.0034 for TMTV_PARS_ and *p* < 0.0001 for TMTV_manual_). Hazard ratios (high-TMTV group vs. low-TMTV group) were 2.1 (range 1.3–3.5) for TMTV_PARS_ and 3.3 (range 2.0–5.6) for TMTV_manual_.

For OS, the area under the ROC curve was 0.66 for TMTV_PARS_ and 0.71 for TMTV_manual_ (Figures 2C,D). The optimal cutoffs for predicting OS were 220.80 ml for TMTV_PARS_ and 327.14 ml for TMTV_manual_. The 5-year OS rates were 68.3 and 39.3% for the low- and high-TMTV_PARS_ groups and 73.0% and 33.9% for the low- and high-TMTV_manual_ groups, respectively (Figures 3C,D). The log-rank test indicated a significantly longer PFS time in the low-TMTV group for both TMTV estimation methods (*p* = 0.0016 for TMTV_PARS_ and *p* = 0.0001 for TMTV_manual_). Hazard ratios (high-TMTV group vs. low-TMTV group) were 2.4 (range 1.4–4.1) for TMTV_PARS_ and 3.1 (range 1.8–5.3) for TMTV_manual_.

### Routine Cohort

Concerning the routine cohort, 430 patients were analyzed; 35% of them had lung cancer, 17% lymphoma, 7% breast cancer, 6% colorectal cancer, 5% melanoma, 5% head and neck cancer, 4% esophageal cancer, and 15% another cancer. In 6% of the cases, the patients were followed up in another center, and we did not have the proven cancer origin.

The median Dice score across all patients between the suspicious PARS ROIs and the manual ROIs was 0.48. The average Dice score was 0.42. For automatic segmentation, median TMTV was 7.37 ml, maximum TMTV was 1,626.97 ml, and minimum TMTVs was 0.00 ml. For manual segmentation, median TMTV was 20.09 ml, maximum TMTV was 4,076.63 ml, and minimum TMTV was 1.00 ml (Table 1 and Supplementary Figure 1). The intraclass coefficient between PARS and manual TMTV was 0.61 (Table 1). Concerning Bland–Altman plot, the deviation from the mean between TMTV_manual_ and TMTV_PARS_ was +60 ml with a confidence interval of −386 to +506 ml (see Figure 1B).

## Discussion

We analyzed an automatic segmentation software prototype using CNN in PET to distinguish hypermetabolic foci suspicious for cancer from nonsuspicious foci in two distinct cohorts of patients.

The first of these cohorts consisted of 119 patients with DLBCL, a disease used for the training of the model and for which the prognostic value of TMTV is well known (10). The median overlapping score of automatic and manual segmentation estimated by the Dice coefficient was 0.65. The ICC between automatically and manually determined TMTVs was 0.68. As follow-up was available for this cohort, survival analysis based on volume thresholds determined by the ROC curves showed that automatically determined TMTVs remained a predictive factor for PFS and OS, but hazard ratios were however lower than for manually determined TMTVs.

The second cohort consisted of 430 patients with a variety of cancers who were referred for PET/CT evaluation. The aim of the analysis of this cohort was to determine the possible utility of the algorithm for clinical routine, in terms of speed and reliability of the analysis of the different foci, and the estimation of the TMTVs. The median overlapping score of automatic and manual segmentation estimated by the Dice coefficient was 0.48. The ICC between automatically and manually determined TMTVs was 0.61.

The scanner type and acquisition parameters were different between the two cohorts. However, the results obtained were relatively similar despite these differences. Moreover, the manual segmentation methods differed (fixed threshold for the clinical research cohort and adaptive threshold for the routine cohort), but this did not greatly influence the results. The use of the 41% SUV_max_ thresholding method has been published in the context of DLBCLs and is a standard in clinical research (15), although much discussed (16). In particular, this method is difficult to use in clinical routine where tumor lesions are often smaller than those observed in DLBCL where a threshold of 41% of the SUV_max_ becomes unsuitable because of the partial volume effect for small lesions (29).

Finally, in the PARS configuration, to limit the computation time without impacting the TMTV measurement, only segmentations with volumes over 1 ml in the routine cohort were analyzed, as potentially small tumors were observed while the limit of 2 ml was used in the research cohort, as DLBCLs present generally large tumors.

In recent years, a number of algorithms have been developed that focus on PET segmentation, mainly in lymphoma, using different branches of artificial intelligence (30–32). In particular, machine learning using CNNs is a major advance in medical imaging. In PET, this technology stands to assist the nuclear physician's interpretation by facilitating, or even refining, the analysis. Concerning lymphomas, and DLBCL particularly, TMTV is usually not calculated during pretherapeutic PET/CT because it takes too long to determine using manual segmentation. Automatic or semiautomatic determination of TMTV could enable clinicians to integrate it in the determination of prognosis and therapeutic adaptation.

PARS is among the first published and validated CNN algorithms for PET/CT lesion classification (21). It was developed to detect FDG foci, and to predict the anatomic location and the expert classification (i.e., suspicious or not suspicious for cancer). It was trained on 380 examinations of patients with lung cancer or lymphoma with a validation set of 126 examinations and a test set of 123 patients (21).

In a recent study (33), the PARS software prototype was tested on a cohort of 280 patients with DLBCL. As with this study, we have established the ability to determine the prognosis of DLBCL using automatic segmentation. The authors however obtained a better lymphomatous lesion recovery coefficient (Dice) of 0.73 and a better TMTV correlation of 0.76. The automatically determined TMTVs were, as in our study, predictive of total and PFS with hazard ratios of 2.8 and 2.4, respectively. The difference in Dice coefficients and TMTV correlation could be explained by the difference in the populations.

Our results are consistent with a recent study (34), in which the performances of a CNN model, based on nnU-Net, were investigated to automatically segment TMTV in patients with DLBCL. A first cohort of 639 patients with pretherapeutic FDG PET/CT was used to train the model. In this cohort, the mean Dice score and Jaccard coefficients for manual and automatic segmentations were 0.73 and 0.68, respectively. There was a mean underestimation of automatic TMTV by 12 ml (*p* = 0.27). An external validation was done on a second cohort of 94 patients. In this testing set, the mean underestimation of automatically determined TMTV was 116 ml, which was statistically significant (*p* = 0.01).

Concerning the clinical routine database, we chose to analyze the examinations of patients followed for any cancerous pathology, whereas the model was trained only on lung cancer and lymphomas. This approach corresponds well to the clinical routine where the pathology is variable, and the results remain consistent with those of the research cohort. Nevertheless, the results are more similar to those obtained for the research cohort, which is closer to the training conditions of the algorithm.

**Figure 4:**
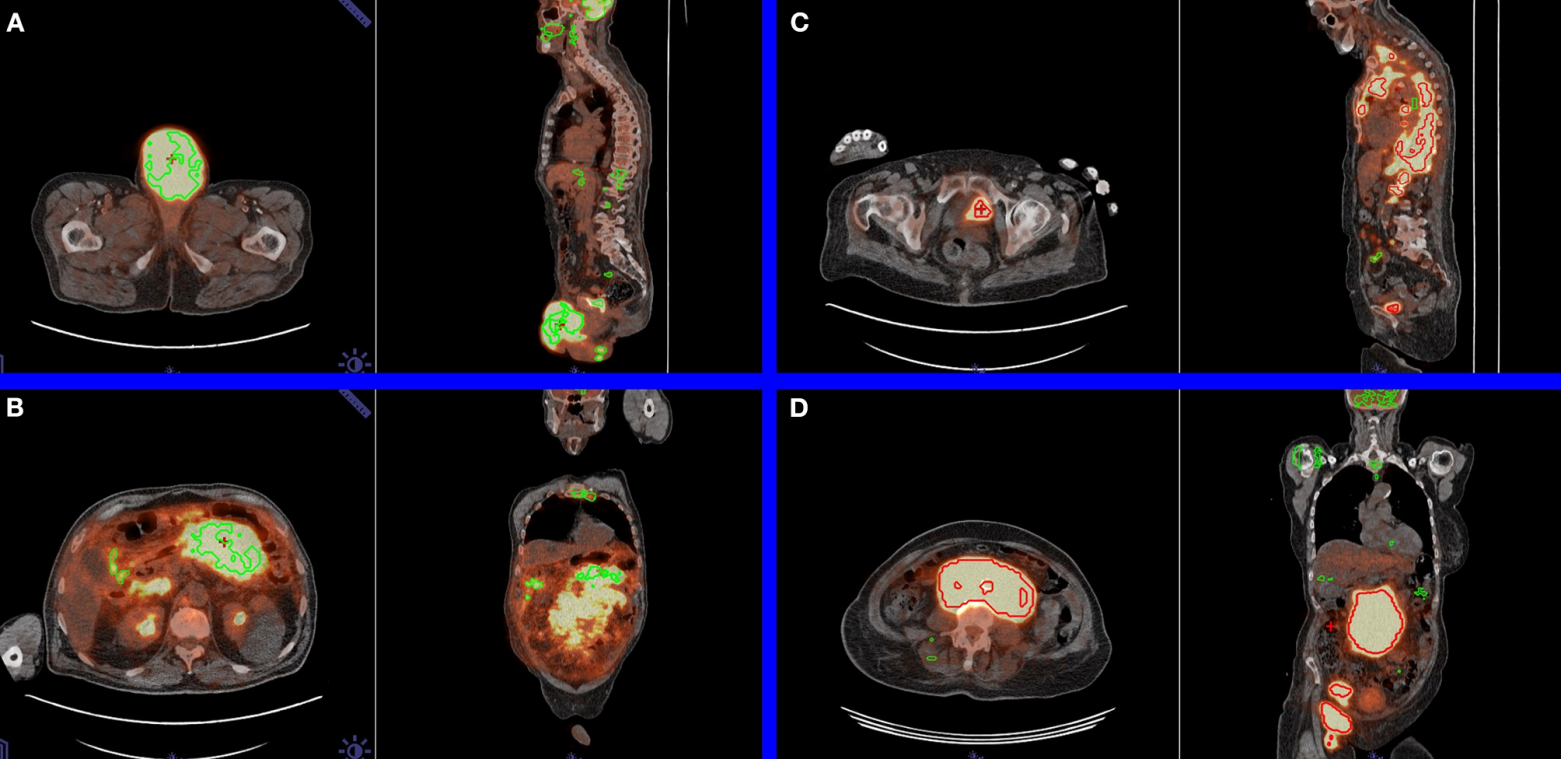
Examples in axial and sagittal views of limitations of the automatic segmentation. **(A)** Pathological testicular mass labeled as physiological by positron emission tomography Assisted Reporting System (PARS) (false negative). For this patient, the manually and automatically obtained total metabolic tumor volumes (TMTVs) were 281.18 and 5.18 ml, respectively. **(B)** Pathological mesenteric mass was erroneously labeled as physiological by PARS (false negative). For this patient, the manually and automatically obtained TMTVs were 2,125.38 and 89.35 ml, respectively. **(C)** Physiological urinary bladder focus was erroneously labeled as pathological by PARS (false positive). For this patient, the manually and automatically obtained TMTVs were 816.94 and 661.08 ml, respectively. **(D)** Pathological mesenteric mass was correctly labeled as pathological by PARS (true positive). For this patient, the manually and automatically obtained TMTVs were 1,369.19 and 1,343.88 ml, respectively.

Although promising, the PARS software prototype tends, in this study, to underestimate the number of cancerous foci, leading to some false-negative cases (see Figure 4). For both clinical research and clinical routine cohorts, the results obtained suggest that a manual check is still needed after the automatic segmentation.

## Conclusion

The purpose of our study was to evaluate the software prototype PARS, which applies CNNs to detect carcinologically suspicious foci of hypermetabolism in FDG PET scans. The total tumor metabolic volumes determined by PARS were predictive of OS and PFS for patients belonging to the DLBCL research cohort. The segmentations and TMTVs determined automatically by the algorithm need to be verified and, sometimes, corrected to be similar to the manual segmentation in both clinical research and clinical routine.

## Data Availability Statement

The datasets generated for this study are available on request to the corresponding author.

## Ethics Statement

The studies involving human participants were reviewed and approved by Henri Becquerel Center Internal Ethics Committee. The patients/participants provided their written informed consent to participate in this study.

## Author Contributions

PD is the guarantor of the paper. PD, PV, RM, and PP designed the study. PD, SB, PP, and MT ensured inclusion and follow-up of patients. PP, FE, MT and PD managed imaging procedures. PP, PD and PV analyzed the data. LS and VS developed the software prototype. All authors contributed in drawing up the manuscript.

## Conflict of Interest

LS and VS are employees of the company Siemens Healthineers.

The remaining authors declare that the research was conducted in the absence of any commercial or financial relationships that could be construed as a potential conflict of interest.

## Supplementary Material

Supplementary Figure 1 Boxplot showing the distribution of the Dice scores for the research cohort (DLBCL) and for the routine cohort (miscellaneous, lung cancer, and lymphoma).
