# Supplementary figures and images for: Evaluation of an Automatic Classification Algorithm Using Convolutional Neural Networks in Oncological Positron Emission Tomography
Source: Front Med (Lausanne). 2021 Feb 26;8:628179. doi: 10.3389/fmed.2021.628179 (PMC7953145; doi:10.3389/fmed.2021.628179)

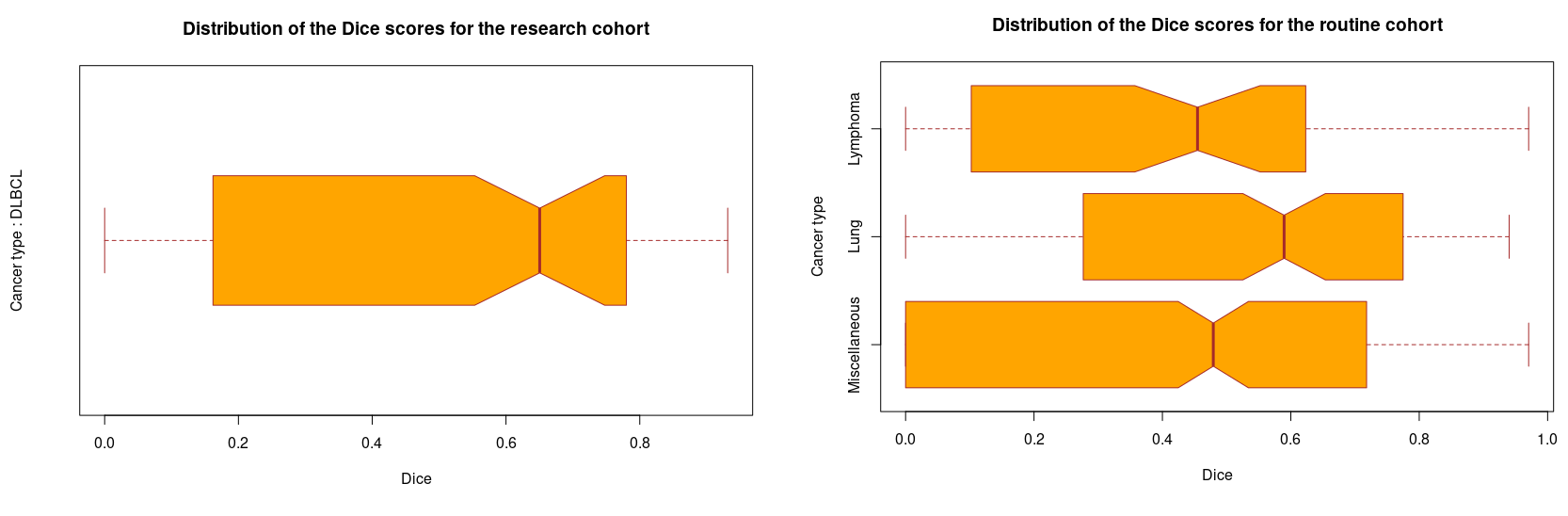

Supplement: Supplementary Figure 1 — Boxplot showing the distribution of the Dice scores for the research cohort (DLBCL) and for the routine cohort (miscellaneous, lung cancer, and lymphoma). [file Image_1.PNG]
